# Molecular features in gangliogliomas: a systematic review

**DOI:** 10.1007/s00381-026-07170-7

**Published:** 2026-02-21

**Authors:** Benedito Jamilson Araújo Pereira, Sueli Mieko Oba-Shinjo, Ivy Karoline Herculano de Azevedo, Yuri Reis Casal, Wen Hung Tzu, Antônio Nogueira de Almeida, Wellingson Silva Paiva, Suely Kazue Nagahashi Marie

**Affiliations:** 1 Laboratory of Molecular and Cellular Biology (LIM15), Department of Neurology, Faculdade de Medicina FMUSP, Universidade de São Paulo, Avenida Dr Arnaldo, 455/4º Andar/Sala 4110, São Paulo, SP Brazil; 2 Hospital das Clínicas HCFMUSP, Divisão de Neurocirurgia, Faculdade de Medicina FMUSP, Universidade de São Paulo, São Paulo, SP Brazil; 3 Pathological Division, Faculdade de Medicina FMUSP, Universidade de São Paulo, São Paulo, SP Brazil

**Keywords:** Ganglioglioma, Molecular, Mutations

## Abstract

**Purpose:**

In the present study, a systematic revision in the Medline was conducted to determine the somatic mutation in gangliogliomas.

**Methods:**

A Medline search for relevant publications up to October 2024 using the key phrase “ganglioglioma mutation” led to the retrieval of 297 studies. This corpus provided the basis for the present review. The records without abstract or descriptions of somatic mutations were excluded. Only records in the English language were considered.

**Results:**

A total of 43 papers were evaluated, reporting a total of 1360 cases of ganglioglioma. Among them, 528 cases presented mutations in 6 genes: *BRAF*^*V600E*^,* FGFR1*,* H3K27M*,* KRAS*,* IDH1*, and* RAF1*. The most common mutation was *BRAF*^*V600E*^ present in 36.94%, followed by the mutation in *FGFR1*, reported in 1.18% of cases.

**Conclusion:**

*BRAF*^*V600E*^ mutation was the most common alteration observed in gangliogliomas, whereas mutations in other genes, such as *FGFR1*, *H3K27M*, *KRAS*, *IDH1*, and *RAF1*, were rare.

## Introduction

Gangliogliomas are rare, well-differentiated neuroepithelial tumors that predominantly occur in children and young adults. These tumors are indolent and are associated with good long-term survival outcomes. Complete surgical resection offers the potential for a cure if the entire tumor is successfully removed [1, 60].

Gangliogliomas represent a significant clinical challenge in pediatric epilepsy and neuro-oncology. Although seizure outcomes in children with gangliogliomas are well documented, comprehensive data on the tumor behavior remain limited [43, 48].

The latest edition of the World Health Organization (WHO) classification of central nervous system (CNS) tumors recognizes a benign form of the lesion (grade 1) with its own characteristics; although there are descriptions about intermediate grade (grade 2) (nonetheless, the authors noted the need for intermediate grade 2 ganglioglioma, although no histological criteria have been established for this category [59]) and malignant (grade 3) gangliogliomas, the latest WHO classification does not describe them. Recent literature proposed the use of the term “atypical” for gangliogliomas with unusual clinical or imaging features, such as atypical locations or infiltrative aspects [18, 101]. Patibandla et al. [71], in your sample, exposed results that support the notion that a substantial portion of pediatric gangliogliomas (GGs) demonstrate an atypical imaging appearance and clinical presentation; these “atypical” tumors seem to be correlated with poorer outcomes and higher recurrence rates [71]. Detailed molecular diagnostics could reclassify the previously reported anaplastic gangliogliomas as other tumors including glioblastomas. Studies prior to the 2021 classification, especially those lacking comprehensive molecular workup should be interpreted in this context.

The large-scale genomic and epigenomic approaches have shed light on the key genetic alterations in various brain tumors. For instance, the *KIAA1549:BRAF* fusion has been described as the most frequent alteration in pediatric low-grade gliomas [45, 76], leading to enhanced activation of the mitogen-activated protein kinase/extracellular signal-regulated kinase (MAPK/ERK) signaling pathway [42, 99]. Additionally, *NTRK* and *RAF1* rearrangements were also associated with the activation of the MAPK/ERK pathway [45, 114]. The *PRKCA-SLC44A1* fusion has been reported in papillary glioneural tumors, a rare mixed neuronal-glial tumor [8], while *PRKCA* missense mutations have been detected in chordoid gliomas [8, 31]. However, gangliogliomas do not exhibit *PRKCA* mutations or mutations commonly found in lower-grade gliomas in adults, such as *TP53*, *ATRX*, *TERT* promoter, *CIC*, or *FUBP1* [11], suggesting that gangliogliomas possess a distinct genetic profile.

Histologically, gangliogliomas are characterized by the presence of a mixture of dysmorphic neurons and glial tumor cells. Recent studies have demonstrated alterations of interleukin-1 receptor/Toll-like receptor pathway (IL-1R/TLR) suggesting an inflammatory response, involving both innate and adaptive immune responses in this type of tumor [78, 85]. Interestingly, these pathways have proved being proconvulsant and ictogenic in experimental models, suggesting the implication of these inflammatory pathways in the pathophysiology of epilepsy, a very common symptom related to ganglioglioma [2, 103].

In the present study, a systematic revision in the Medline was conducted to determine the somatic mutation profile in gangliogliomas.

## Methods

A Medline search up to August 2024 using the key phrase “ganglioglioma mutation” returned 297 papers. The records without abstract or descriptions of somatic mutations were excluded. Only records in the English language were considered, and GG was the only pathological diagnosis for the evaluation of the tumor.

**Fig. 1:**
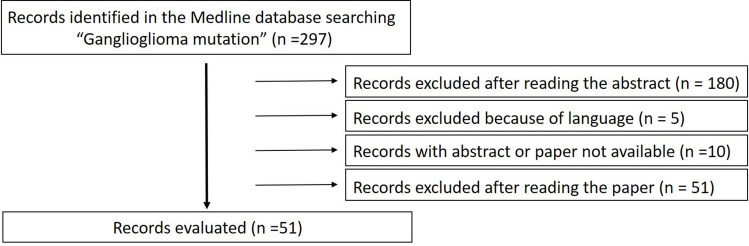
PRISMA (preferred reporting items for systematic reviews and meta-analyses) study flow diagram

A total of 43 papers about somatic mutations (all included cases were evaluated for mutation detected at the DNA level or for the presence of the specific protein BRAF^V600E^) (see Fig. 1 for the preferred reporting items for systematic reviews and meta-analyses (PRISMA) study flow diagram) fulfilled the above criteria. Molecular data were retrieved and the frequency of mutations and miR alterations were analyzed.

References from all articles selected were further examined to identify additional suitable studies and possible patient duplication. Of these retrieved articles, 197 were excluded because the abstracts were not in English or the articles did not meet the inclusion criteria. The remaining 100 articles were then carefully analyzed, and an additional 57 were excluded due to either a lack of confirmed histopathological diagnosis of ganglioglioma or insufficient detail regarding the molecular methods used to determine the mutation profile. Ultimately, 43 studies (see Fig. 1 for the PRISMA study flow diagram) encompassing a total of 1360 cases were included in this analysis of the somatic mutation profile in gangliogliomas.

## Results

A total of 1360 cases from studies across all continents were evaluated, comprising 621 males and 480 females, with a male-to-female ratio of 1.29:1. Sex was unreported for 259 cases (Table 1).

**Table 1:** Literature review

Author	Year	Country	N	M	F	Age	Method	*BRAF*^*V600E*^	*FGFR1*	*H3K27M*	*KRAS*	*IDH1*	*RAF1*
**Schindler** [89]	2011	Germany	83	nr	nr	nr	Sanger	17	NI	NI	NI	NI	NI
**Horbinski** [39]	2011	USA	98	54	44	46.12	Real time PCR, melting, IHC	NI	NI	NI	NI	8	NI
**Myung **[68]	2012	Korea	51	22	29	25	Sanger	12	NI	NI	NI	NI	NI
**Dahiya **[16]	2013	USA	47	33	20	12	IHC	18	NI	NI	NI	NI	NI
**Koelsche** [50]	2013	Germany	71	37	34	22	Sanger, IHC	60	NI	NI	NI	NI	NI
**Prabowo** [79]	2013	Netherlands	93	48	45	24.6	Sanger, IHC	38	NI	NI	NI	NI	NI
**Donson** [19]	2014	USA	24	9	15	8.83	Sanger, IHC	13	NI	NI	NI	NI	NI
**Gupta** [32]	2014	USA	29	13	14	10.4	Sanger	7	NI	NI	NI	NI	NI
**Martioni** [64]	2015	Italy	6	4	2	26.1	Targeted sequencing	4	NI	NI	NI	NI	NI
**Gessi** [29]	2015	Germany	25	15	10	20.5	Pyrosequencing, IHC	2	NI	NI	NI	NI	NI
**Breton** [7]	2016	France	50	32	18	16.2	Sanger, ASQ-PCR, IHC	32	NI	NI	NI	NI	NI
**Zanello** [112]	2016	France	18	11	7	37.7	MALDI-TOF	9	3	NI	NI	NEG	NI
**Liu** [55]	2016	Japan	3	3	0	3.3	Sanger, IHC	3	NI	NI	NI	NEG	NI
**Cicuendes** [15]	2016	Spain	1	1	0	18	Real time PCR, IHC	1	NI	NI	NI	NI	NI
**Qaddoumi** [81]	2016	USA	17	nr	nr	nr	WGS, WES	6	NEG	NI	NI	NI	NI
**Wang** [104]	2016	USA	1	0	1	35	Sanger	1	NI	NI	NI	NI	NI
**Joyon** [46]	2016	France	2	1	1	13.5	Sanger, IHC	2	2	NI	NI	NI	NI
**Fiset** [27]	2016	Canada	1	0	1	4	IHC	1	NI	NI	NI	NI	NI
**Mesturoux **[66]	2016	France	12	6	6	28.83	RT-PCR	3	NI	NI	NI	NEG	NI
**Kakkar** [47]	2017	India	64	18	10	23.6	Sanger, IHC	6	NI	NI	NI	NI	NI
**Zhang** [115]	2017	China	24	15	9	19.9	Sanger	8	NI	NI	NI	NI	NI
**Pagès** [69]	2018	France	54	30	24	8	Sanger, MALDI-TOF, IHC	27	5	NI	NI	NI	NI
**Miller** [67]	2018	USA	1	0	1	13	WGS, WES	1	NI	NI	NI	NI	NI
**Pekmezci** [72]	2018	USA	40	23	17	21	Targeted sequencing	18	NI	5	2	NI	1
**Ramaglia** [82]	2019	Italy	18	nr	nr	nr	Sanger	11	NI	NI	NI	NI	NI
**Trisolini** [100]	2019	Italy	75	41	41	34	Sanger	14	NI	5	NI	NI	NI
**HO** [36]	2020	USA	8	2	5	16.4	Pyrosequencing	5	NI	NI	NI	NI	NI
**Yau** [110]	2020	AUS	1	1	0	32	Targeted sequencing	1	NI	NI	NI	NI	NI
**Durselwicz** [22]	2020	Poland	3	3	0	40.3	ASQ-PCR, IHC	2	NI	NI	NI	NI	NI
**Kraus** [52]	2020	AUT	1	1	0	28	Targeted sequencing	NI	1	NI	NI	NI	NI
**Petralia** [75]	2020	USA	18	nr	nr	nr	WGS	8	NI	NI	NI	NI	NI
**Ehrestdet** [24]	2020	Sweden	27	nr	nr	nr	IHC	13	NI	NI	NI	NI	NI
**Solomon **[94]	2021	USA	1	1	0	7.5	Single Gene sequencing	1	NI	NI	NI	NI	NI
**Makino** [63]	2021	Japan	1	nr	nr	nr	Sanger	NI	NI	NI	NI	1	NI
**Wang** [106]	2021	China	30	18	12	16.5	Targeted sequencing	20	NI	NI	NI	2	NI
**Kim** [49]	2021	Korea	3	3	0	10.5	Targeted sequencing	3	NI	NI	NI	NI	NI
**Forest** [28]	2021	France	22	1	8	24.5	NGS snapshot	3	NI	NI	NI	NI	NI
**Dyke** [23]	2022	Australia	21	10	11	22.5	MLPA	9	NI	NI	NI	NI	NI
**Xie** [108]	2023	China	208	132	76	20	IHC	54	NI	NI	NI	NI	NI
**Liu** [56]	2023	China	51	33	18	49	Real time PCR	26	NI	NI	NI	NI	NI
**Hoffman** [38]	2023	Germany	72	nr	nr	nr	WES	35	NI	NI	7	NI	NI
**Dang **[17]	2023	USA	1	0	1	13	WES, targeted sequencing	NI	1	NI	NI	NI	NI
**Aufret** [3]	2024	France	9	nr	nr	nr	WES, targeted sequencing	NI	4	NI	NI	1	NI
**Number of cases with mutation**								494/1279	16/102	10/115	9/98	12/120	1/40
**Relative number of mutation (%)**								**38.63**	**15.68**	**8.69**	**9.18**	**10**	**2.5**
**Total number of cases with mutation (%)**			**528**					**36.32**	**1.18**	**0.74**	**0.66**	**0.88**	**0.07**
**Total number of cases analyzed**			**1360**					X					

*NI* not investigated, *NEG* investigated but negative in specimen

### Age

The confirmed ages of the included patients at surgery (mean age) ranged from 1 year to 46.12 years (mean 24.6 ± 11.86 years old).

### Molecular methods

The mutational status was determined by distinct methods, including the detection of the specific protein BRAF^V600E^ through immunohistochemistry (4 reports) and by DNA sequencing using the Sanger method (16 reports), allele-specific quantitative PCR (2 reports), real-time PCR (2 reports), pyrosequencing (2 reports), massarray iPlex (MALDI-TOF, 2 reports), and multiplex ligation-dependent probe amplification (MLPA, 1 report) and by sequencing cDNA by reverse transcription PCR (1 report). More recent publications (2015–2018) used high-throughput sequencing by targeted sequencing, whole exome sequencing (WES), and whole genome sequencing (WGS).

### Mutations

In total, mutations were found in 6 genes: *BRAF*^*V600E*^(36.32%),* FGFR1 *(1.18%),* H3K27M *(0.74%),* KRAS *(0.66%),* IDH1 *(0.088%), and* RAF1 *(0.07%) were described in gangliogliomas (Table 1). The next-generation sequencing approaches including targeted sequencing, WES, and WGS have expanded the ability to detect a range of *BRAF* gene alterations beyond the well-known hotspot mutation *BRAF*^*V600E*^. These advancements have made it possible to identify additional variants, such as *BRAF* duplication [67], *BRAF* novel in-frame insertion [72], *BRAF* fusions [72], *NF1* mutation [17, 72], combination of *BRAF*^*V600E*^ with *CDKN2A* deletion [72, 106], and other gene fusions such as *ABL2-GAB2* and *ERC2-RAF1* [72].

**Fig. 2:**
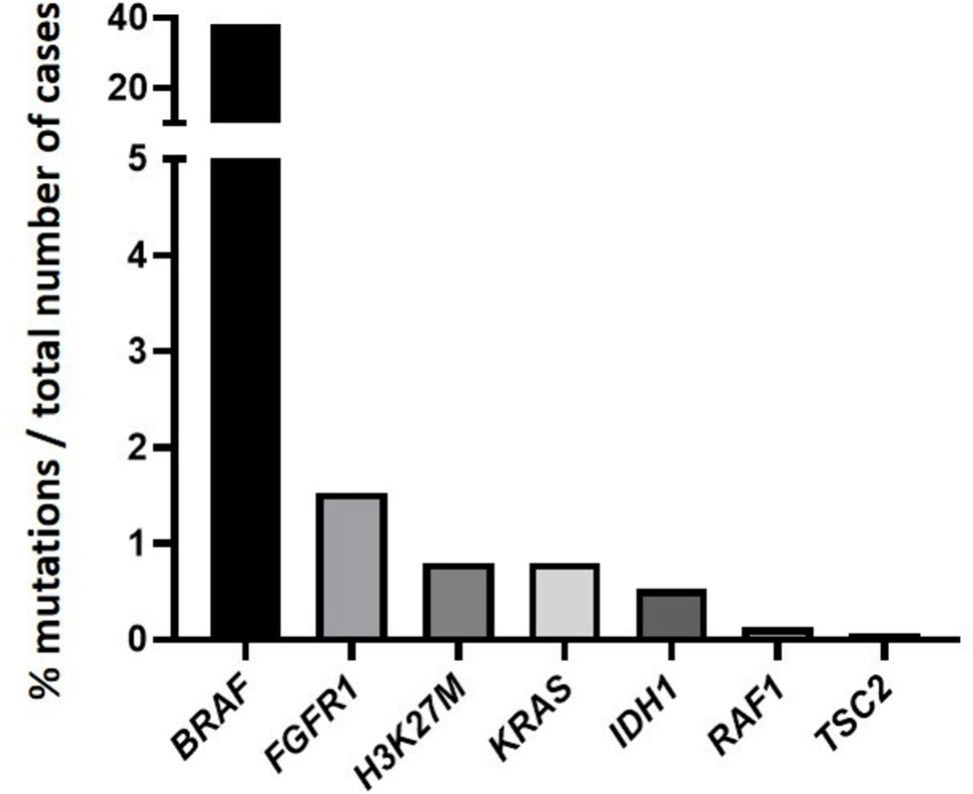
Frequency of the most common genetic alterations found in gangliogliomas according to independent studies

**Fig. 3:**
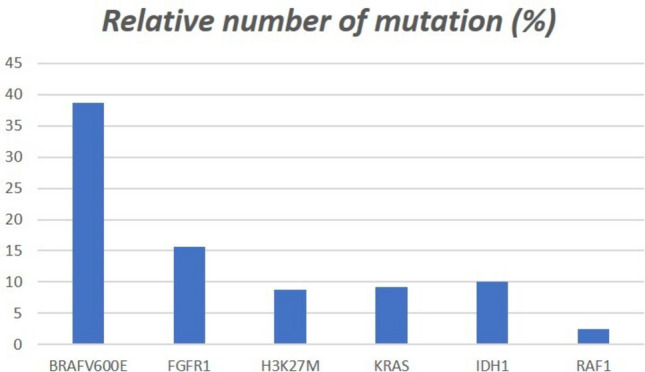
Relative number of mutation (%)

The number of ganglioglioma cases and the frequency of the mutations (Figs. 2 and 3) are discussed in details below.

## Discussion

The objective of carrying out research such as this, despite the limitations that will be mentioned later, is to expand knowledge about these tumors, with regard to the study of the molecular profile; drawing parallels with other tumors of the central nervous system, which, as observed in the latest classification of the World Health Organization (WHO 2021) [59], several tumor types already include molecularly defined subtypes, such as medulloblastomas, or use advances in this field to better define prognosis and optimize therapies, such as gliomas [98]; other tumors, such as meningiomas, although they do not yet present changes in their classification or treatment, are making great strides towards this with research investigating new markers for more aggressive forms [73] or even proposing new classifications [88, 105]. Gangliogliomas, however, have not shown comparable advances in this same period, which suggests this is a segment in need of further exploration.

Gangliogliomas harbor mutations in the B-Raf proto-oncogene serine/threonine kinase (*BRAF*), particularly the mutation *BRAF*^V600E^, where the glutamic acid-to-valine substitution within the active site of the encoded serine/threonine kinase domain leads to its constitutive activation that promotes an oncogenic transformation. Initially, the *BRAF*^V600E^ mutation was identified in 18% of gangliogliomas by traditional sequencing methods [89]. With more sensitive techniques, detection rates increased to 35.96%, confirming *BRAF*^V600E^ as the most frequent mutation in these tumors (Fig. 2). However, *BRAF*^V600E^ is not specific to gangliogliomas; it has also been described in pilocytic astrocytoma, dysembryoplastic neuroepithelial tumor, pediatric IDH-wildtype diffuse astrocytoma, polymorphous low-grade neuroepithelial tumor of the young, pleomorphic xanthoastrocytoma, and epithelioid glioblastoma [14, 20, 40, 51, 81, 89, 114], as confirmed in a screen of 1320 CNS tumors [89]. Moreover, the alterations in *BRAF* signaling pathways have been implicated in the pathogenesis of gliomas in children [45, 76].

To facilitate molecular stratification of tumors, a BRAF^V600E^ mutation-specific monoclonal antibody was developed [12], with 97% sensitivity and 98% specificity for detecting this mutation across different tumor types [57]. In gangliogliomas, *BRAF*^V600E^ sequencing results were highly concordant with immunohistochemistry (IHC) (up to 94%), and 58% BRAFV600E positivity has been reported by IHC [50]. Clinically, *BRAF*^V600E^ immunopositivity has been associated with poorer recurrence-free survival in colon carcinoma [92]. In gangliogliomas, prognosis is also influenced by the extent of resection [43, 61, 62], tumor location, and seizure history [25].

The main factors in the evolution and natural history of gangliogliomas (GGs) are related to location, epilepsy control, and tumor prognosis (recurrence and death). The most common location is the temporal lobe, and GGs occur more frequently in children and young adults with early-onset focal epilepsy [5, 6]. They are regarded as a common seizure-associated tumor, accounting for approximately 40% of all epileptogenic tumors [33, 113].

Among the mutations studied, the most consistent finding in the literature and in this review is *BRAF*^V600E^. Clinically, this mutation shows several correlations: it was more frequent in non-recurrent cases; progression-free survival did not differ significantly by *BRAF*^V600E^ status [68];* BRAF*^V600E^ positive staining was associated with worse recurrence-free survival [16]. Patients with BRAF-mutated GGs were significantly younger at surgery (median 19 years, range 5–52) compared to wild-type tumors (median 31 years, range 12–69). Contingency analysis showed no significant difference in the percentage of brainstem versus non-brainstem GGs harboring *BRAF*^V600E^ in that limited cohort [19]; Gupta et al. found that seven of sixteen (44%) tumors with features of classic ganglioglioma carried a *BRAF*^V600E^ mutation, linking infratentorial morphologic groups to typical cerebral GGs [32]; Martinoni et al. reported that all but one patient (83.3%) achieved Engel Class Ia seizure control, whereas the single patient with a concomitant BRAF mutation achieved Engel Class II [64]. Furthermore, in previous experience, *BRAF*^V600E^ mutation is strongly associated with temporo-mesial low-grade epilepsy-associated tumors LEAT [65]*.*

BRAF inhibitors (BRAFi) are small kinase inhibitors that bind selectively to V600E mutated B-Raf proteins and stop them from activating MEK, thus inhibiting the MAPK/ERK signaling cascade, preventing aberrant cell signaling [91]. Despite advances, intrinsic resistance to BRAFi remains a challenge, often mediated by receptor tyrosine kinase upregulation, PTEN loss, or a pro-angiogenic tumor microenvironment [74]. In melanoma, BRAFi treatment produces reversible tumor regression [37]. Vemurafenib (Zelboraf), dabrafenib (Tafinlar), and encorafenib (Braftovi) are FDA- and EU-approved for unresectable or metastatic melanoma [21, 91]. These drugs are now under evaluation in pediatric cancers, including brain tumors. They represent a potential novel therapy for BRAF-mutant GGs refractory to surgery and standard chemotherapy [77]. Responses have also been reported in children: Brown et al. described successful treatment of recurrent anaplastic PXA with *BRAF*^V600E^ mutation using dabrafenib [9]. A 2-month-old infant with *BRAF*^V600E^ mutated hypothalamic chiasmatic glioma responded to dabrafenib [53]. In a phase I study of dabrafenib in pediatric patients with BRAF-mutated LGG, HGG, and other solid tumors, 75% of HGG patients responded completely or partially, and 14/15 LGG patients showed partial response or stable disease [34]. Phillipe et al. [77] provided further evidence that clinical and radiological responses can be rapidly achieved in brainstem GGs harboring *BRAF*^V600E^. However, abrupt discontinuation of dabrafenib induced rapid relapse in three pediatric patients, despite 24 months of clinical stability under therapy.

The H3K27M mutation was detected in 0.74% of gangliogliomas (Fig. 2). Mutations in histone H3 variants H3.3 and H3.1 with somatic gain-of-function were classically reported in midline diffuse pediatric gliomas and a subset of pediatric supratentorial high-grade gliomas [30, 90, 107]. The *H3K27M* substitution in H3F3A or HIST1H3B/C is associated with aggressive diffuse midline gliomas, especially in pediatric patients [10, 95, 97], while the G34R/V mutation in H3F3A has been described in hemispheric glioblastomas [97].

Until recently, it was unclear whether gangliogliomas consisted of glial, neuronal, or both cell types [50]. Detection of BRAFV600E and H3K27M positivity in both glial and neuronal components in dual-mutated cases confirmed their mixed glioneuronal nature [69].

Mutations affecting histone H3, particularly H3F3A K27M, are a hallmark of diffuse midline gliomas [69] but are increasingly recognized in other low-grade CNS tumors. Diffuse midline gliomas with H3 K27M mutation are rare, typically arising in midline structures and the brainstem of younger patients [90, 97], and carry a devastating prognosis [58]. Epigenome-wide methylation profiling has confirmed them as a distinct methylation class [97].

A few reports described co-occurring H3F3A K27M and *BRAF*^V600E^ mutations in midline low-grade GGs. Limited data suggest H3F3A K27M should be assessed in all GGs, as it may predict progression, anaplastic transformation, metastasis, and poor prognosis [46]. Conversely, circumscribed anaplastic GGs with H3F3A K27M may have a more favorable prognosis than infiltrative counterparts [80].

Recurrent mutations in H3F3A and HIST1H3B (encoding histones H3.3 and H3.1) have also been described in pediatric and adult HGG and confer poor prognosis [97, 102, 107]. These mutations show anatomical segregation: H3F3A K27M is found in the thalamus, pons, and spinal cord; HIST1H3B K27M in diffuse intrinsic pontine gliomas; and H3F3A G34R/V in the cerebral hemispheres [97, 102, 107].

Gangliogliomas also show mutations in FGFR1/FGFR2, observed in 1.18% of cases (Fig. 2). Such alterations are reported in low-grade gliomas, melanoma, and glioblastoma [54, 83]. FGFR1 amplification with constitutive activation of downstream signaling has been identified in pediatric low-grade gliomas [96, 114], adult midline gliomas [72], and forebrain development [35]. FGFR1 mutations were also reported in pilocytic astrocytoma [4, 44] and, at unexpectedly high frequency, in adult optic pilocytic astrocytoma not associated with NF1 [100], possibly reflecting its role in midline commissure formation [93].

Pekmezci et al. reported that FGFR- and RAF1-altered gangliogliomas localized to cerebral hemispheres. Three cases with oligodendroglial-like morphology harbored FGFR alterations, while two others with astrocytic morphology also showed FGFR changes. No significant differences in event-free survival were observed between FGFR- and RAF1-altered tumors [72].

The mutation in isocitrate dehydrogenase type 1 (*IDH1*) gene was first described in glioblastoma [70] and is common in adult diffuse gliomas [109], but it is rare in GGs (0.88%) [39]. Horbinski et al. reported IDH1-mutated GGs outside the temporal lobe (location unknown in one case). Four of eight were atypical but not grade III, and one was grade III at diagnosis. IDH1 mutation was associated with adverse outcomes including malignant transformation and death, and on multivariate analysis was the strongest prognostic factor after age [89]. Older reports of “IDH1-mutant gangliogliomas” must be interpreted cautiously, as they may not conform to current diagnostic definitions.

Mutations in the KRAS proto-oncogene, GTPase gene (*KRAS*), are rare in gangliogliomas, being described in only 12 out of 143 gangliogliomas [72] (0.66%) (Fig. 2 and Table 1). *KRAS* mutations are often observed in younger patients [13]. Although KRAS mutations have been linked to VEGF upregulation [26], no evidence of increased vascularity (e.g., gadolinium enhancement, arteriovenous shunting) was found in KRAS-mutant GGs in this review. The *KRAS*^G12D^ variant has been associated with gliosis [87].

Mutations in the Raf-1 proto-oncogene, serine/threonine kinase gene (*RAF1*), have been described in gangliogliomas; however, they were rare (0.07%) (Fig. 2). RAF1 plays a role in proliferation and survival, and fusions with partners such as TRIM33, FYCO, QKI, NF1A, SRGAP3, and ATG7 activate MAPK signaling [26, 59, 83]; their role in pediatric brain tumors remains unclear [41]. RAF1 fusions typically involve the N-terminal domain, where dimerization with partner proteins is essential for oncogenesis. Combination therapy with MEK inhibition has shown responses in pediatric low-grade gliomas [84, 111]. Therefore, targeted strategies that block RAF1 dimerization may be required [86].

## Conclusion

The BRAF^V600E^ mutation was the most frequent in gangliogliomas, whereas mutations in other genes, such as *FGFR1*, *H3K27M*, *KRAS*, *IDH1*, and *RAF1*, were rare.

## Limitations

Despite its relatively large sample size, this study has certain limitations. Most included articles focused on only one or a few specific mutations, potentially leaving other alterations unreported or underestimated—an important limitation to acknowledge. Different methods have been used in the literature to detect genetic alterations. These techniques vary in sensitivity and specificity, which may have biased the true estimation of mutation frequencies. Considering that NGS is more sensitive for detecting mutations present at low copy numbers, and that this methodology was used in only six studies [3, 17, 38, 67, 75, 81], covering 118 cases (8.7% of the total), it is plausible that the mutation frequency is underestimated. A more accurate molecular profile of GG can be determined with the cumulative inclusion of cases analyzed by NGS. Information was often lacking regarding whether cases showed BRAF alterations alone, BRAF alterations with additional mutations, or whether the presence of single versus multiple mutations influenced survival outcomes. Nevertheless, systematic data collection helped minimize bias and reduce errors inherent to individual institutions, thereby increasing the statistical power of the findings. This approach enhanced the value of results from each participating center and allowed for a more accurate characterization of reported gangliogliomas.

## Data Availability

PubMed research.
